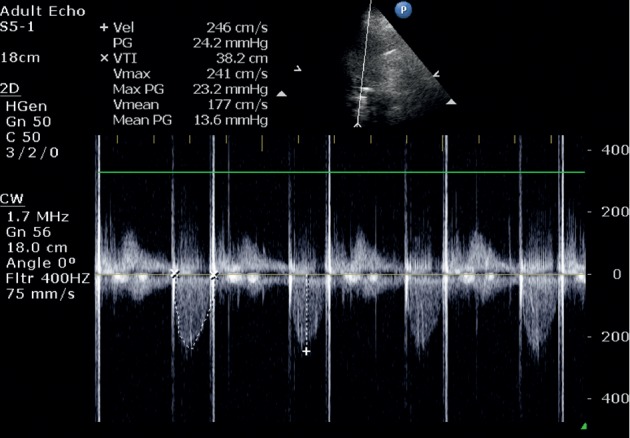# Ankylosing Spondyloarthritis Resulting Severe Aortic Insufficiency and Aortitis: Exacerbation of Ankylosing Spondyloarthritis and Stenosis of the Main Left Coronary Artery after Mechanical Aortic Valve Implantation with Cardiopulmonary Bypass

**DOI:** 10.1155/2020/9538527

**Published:** 2020-01-03

**Authors:** Agnė Balčiūnaitė, Algimantas Budrikis, Eglė Rumbinaitė, Jurgita Sabaliauskienė, Vaiva Patamsytė, Vaiva Lesauskaitė

**Affiliations:** ^1^Department of Cardiac, Thoracic and Vascular Surgery, Medical Academy, Lithuanian University of Health Sciences, Kaunas, Lithuania; ^2^Department of Cardiology, Medical Academy, Lithuanian University of Health Sciences, Kaunas, Lithuania; ^3^Department of Rheumatology, Lithuanian University of Health Sciences, Kaunas, Lithuania; ^4^Institute of Cardiology of the Medical Academy, Lithuanian University of Health Sciences, Kaunas, Lithuania

## Abstract

Ankylosing spondyloarthritis (AS) is a chronic inflammatory rheumatic disease, strongly related to human leukocyte antigen (HLA)-B27. Cardiac involvement in AS manifests in 2 to 10% of patients as aortic insufficiency, aortitis, mitral valve fibrosis, or disturbance in the conduction of the heart. In this article, we present a case of a 49-year-old male patient with AS, who was referred to our medical institution for elective aortic valve surgery because of severe aortic regurgitation. The histological findings revealed fibrosing endocarditis of aortic valve and nonspecific aortitis of aortic root. Late postoperatively, we observed exacerbation of AS and narrowing of the main left coronary artery (LAD). Our case highlights the importance of proper treatment of AS before and after cardiac surgery. Furthermore, in this case, we suspect association between cardiopulmonary bypass, activity of AS, and coronary artery disease.

## 1. Introduction

Cardiac involvement in ankylosing spondyloarthritis (AS) is an uncommon condition, which manifests in 2 to 10% of patients. Since 1930s, when aortitis was initially found in a group of patients with spondylitis, other heart conditions (aortic regurgitation, conduction defects, and cardiomyopathy) were described as associated with AS. Human leukocyte antigen- (HLA-) B27 is presented in the majority of patients with AS [1, 2]. We report a case of a 49-year-old male, who was referred to our medical institution for elective aortic valve surgery and who suffered from poorly controlled AS.

## 2. Case Presentation

### 2.1. Clinical Presentation

The patient was referred to our clinic. Upon arrival, patient denied any cardiac-related symptoms, but he had a history of chest pain during physical activity. Severe aortic regurgitation and dilatation of left ventricle were diagnosed 3 months earlier incidentally, when he was treated at a regional hospital because of ST elevation anterior myocardial infarction. Percutaneous coronary intervention on the left anterior descending artery was performed. Patient denied the history of arterial hypertension or diabetes mellitus, but he was hospitalized and treated due to reactive arthritis in 2007; a positive HLA-B27 was detected at that time. AS was established 6 years ago, and based on complaints, positive HLA-B27 and MRI of the pelvis showed bilateral sacroiliitis. Disease was poorly controlled. Patient used nonsteroidal anti-inflammatory drugs constantly and sulfasalazine for exacerbations. He suffered from spinal stiffness and pain and bilateral knee arthritis during hospitalization. Cardiac auscultation revealed diastolic murmur, predominantly in the aortic area. Mobility of the spine was limited due to stiffness in the lower back, and knee joints were swollen. No other objective significant changes were found.

### 2.2. Diagnostic Tests

**Figure 1:**
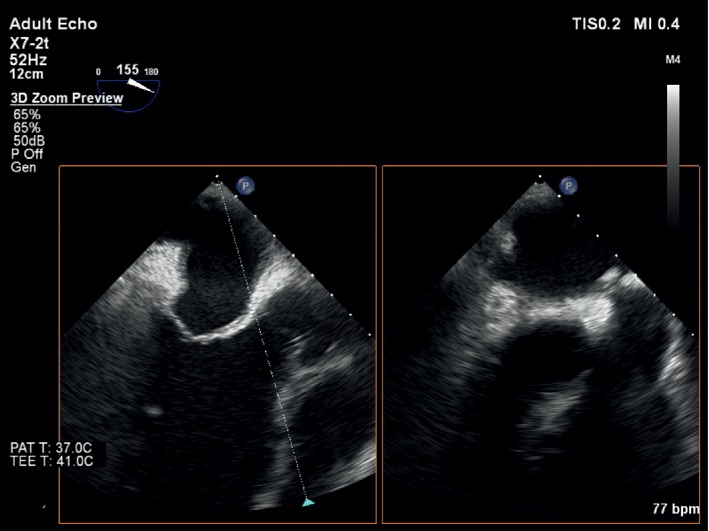


**Figure 2:**
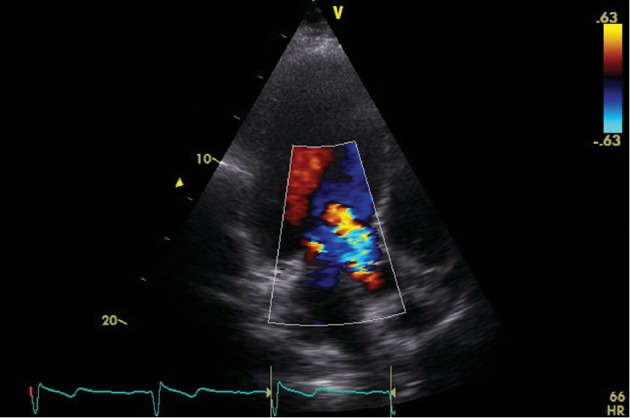


The electrocardiogram (ECG) showed sinus rhythm and negative *T* waves in I and aVL leads. Transthoracic (TTE) and transoesophageal echocardiograms (TEE) presented an enlarged left ventricle (LV) (LV end-diastolic diameter = 71 mm.), resting LV ejecting fraction (EF) = 50%, thickened aortic root wall, insignificant aortic ring sclerosis, thin aortic valve leaflets, and fibrotic margins of right and noncoronary leaflets; all leaflet movements were restricted. The anterior leaflet of the mitral valve was thickened, but the function of mitral valve has not changed significantly (Figure 1). TTE and TEE showed severe aortic regurgitation through aortic valve commissures and central part of the aortic valve (Figure 2). No significant other valve diseases were found. Laboratory examination showed mild anaemia (haemoglobin 125 g/L). Creatinine level, electrolytes, glycaemia, and coagulation parameters were within reference limits. C-reactive protein (CRP) was 54 mg/L. Patient suffered from mixed hyperlipidaemia.

### 2.3. Treatment

**Figure 3:**
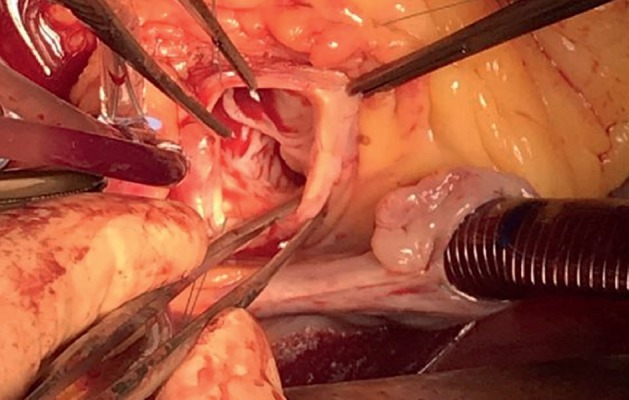


**Figure 4:**
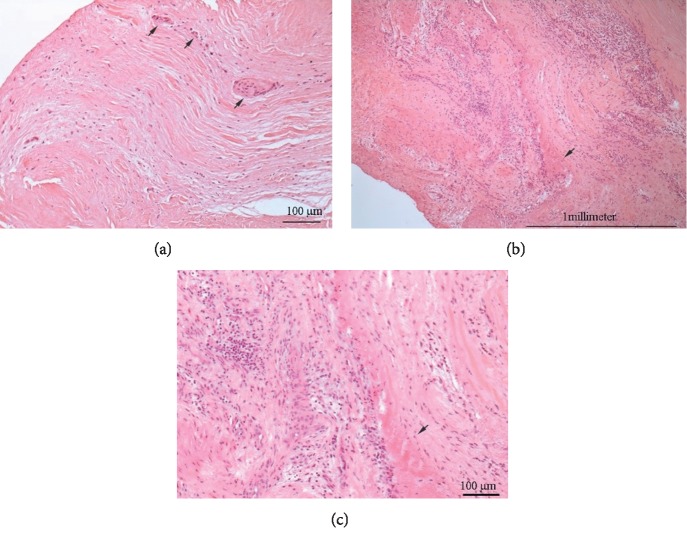


Mechanical aortic prosthesis implantation was suggested by the heart team. Using the cardiopulmonary bypass (CPB) surgical procedure was done. Unusual changes in the aorta were observed during the operation (Figure 3). The adventitia of the proximal part of the ascending aorta was reddened, and the wall was thickened. Aortic valve leaflets were thickened near the basis and restricted. Fibrosis of the basis of the anterior mitral leaflet was detected. After removing aortic cusps, the mechanical aortic valve (Sorin Carbomedics Standard 23 mm.) has been implanted using three 2-0 Prolene continuous sutures. Specimens were taken for pathohistological investigation. The histological findings revealed fibrosing endocarditis of aortic valve and nonspecific aortitis of the aortic root (Figures 4(a)–4(c)). The patient was consulted by a rheumatologist, and diagnosis of ankylosing spondylitis (bilateral sacroiliitis, oligoarthritis, aortitis, and valvulitis) was confirmed. Methylprednisolone 32 mg/day was tapered to a maintenance dose of 8 mg in two weeks.

Postoperative echocardiography (Figure 5) revealed good aortic valve mechanical prosthesis function, slightly reduced LV EF, and no important other valves' pathology. The patient was discharged from the hospital in good condition.

### 2.4. Postdischarge Events

7 weeks later, the patient was hospitalized to the Department of Rheumatology due to increased inflammatory markers (CRP: 398.66 mg/L) in order to differentiate this condition between prosthesis-related infection and exacerbation of AS. Our patient was suffering from inflammatory lumbar pain and arthritis of knee joints. Modified Schober test was 3 cm, lateral lumbar flexion was 7 cm, tragus to wall distance was 18 cm, cervical rotation was 65 degrees, normal hips range of motion, and both knees were swollen and had 10 degrees flexion contractures. Echoscopy of the knee joints showed a synovitis (BASDAI: 7.1).

Patient was treated with antibiotics, firstly with cefazolin and metronidazole 10 days, and then vancomycin was prescribed for 8 days. CRB decreased to 92 mg/L. After intensive investigation including positron emission tomography (PET), it was concluded that the inflammatory parameters increased due to the exacerbation of AS after cardiac surgery. Sulfasalazine 1 g 2 times per day and diclofenac 50 mg 3 times per day were prescribed. Inflammatory markers decreased, and the patient was discharged in good condition from the hospital again.

4 weeks later, the patient was hospitalized due to the new onset of unstable angina pectoris. Urgent coronary angiography was performed, and 95% LAD stenosis was observed. Percutaneous coronary intervention on LAD was performed with good result.

## 3. Discussion

AS is a chronic inflammatory rheumatic disease, belonging to the group of seronegative spondyloarthropathies. Mainly, it involves sacroiliac joints, spine, and peripheral joints; however, it has a well-known association with extra-articular tissues, such as skin, eyes, heart, and mucous membrane. HLA-B27 is strongly associated with seronegative spondyloarthropathies, including AS [1–3]. Cardiac involvement generally manifests itself as aortic insufficiency, aortitis, or disturbance in the conduction of the heart [2, 4]. Mitral insufficiency is determined less frequently, when fibrosis of subaortic tissues reaches the anterior mitral valve leaflet [2, 3]. The cellular inflammation causes proliferative endarteritis which results in fibrotic tissue thickening and aortic root dilatation. Then, the process reaches the aortic annulus, and it causes the basal thickening of the cusps which finally leads to aortic regurgitation [1, 5–8]. The duration of AS is associated with the appearance of aortic regurgitation in patients; after 10 years of AS, it appears for 2%, and after 30 years, it appears for 12% of patients [4]. Bergfeldt reported that HLA-B27 associated inflammatory process was present in 15 to 20% of patients with aortic regurgitation, while there was no significant link between HLA-B27 and aortic regurgitation without rheumatic disease [9]. Conduction disorders (atrioventricular (AV) and intraventricular blocks) in patients with AS are caused by the damage to the arterial supply to the AV node or by inflammation that results in myocardial fibrosis and damages the interventricular septum. Conduction disturbances are the most common cardiac symptoms in AS patients and usually precede other cardiac symptoms [2]. In the cross-sectional study Forsblad-d'Elia et al. reported that 10–33% of patients with AS had conduction disorders which mostly consisted of the 1^st^ degree AV block and prolonged QRS duration, 7 patients had complete bundle branch blocks, and 1 patient had a pacemaker [10].

It is crucial to mention that differential diagnosis of aortitis is challenging, due to the non-specific constitutional symptoms of the patients [11, 12]. This leads to diagnostic dilemmas and might lead to a series of unnecessary diagnostic workups. In our case, the 49-year-old male did not have any aortitis-related symptoms. Severe aortic regurgitation and dilatation of left ventricle was diagnosed 3 months earlier incidentally, and it was known that AS was diagnosed before four years. The most common causes of aortitis among the rheumatic diseases include giant cell arteritis, Takayasu arteritis, infective aortitis, Cogan syndrome (interstitial keratitis, iritis, conjunctival or subconjunctival hemorrhage, fever, and aortic insufficiency), and relapsing polychondritis and spondyloarthritis [11]. The underlying microorganisms of infective aortitis were most often *Staphylococcus*, *Enterococcus*, *Streptococcus,* and *Salmonella* species. Aortitis due to *Mycobacterium tuberculosis* or *Campylobacter fetus* have been reported. Syphilitic aortitis is also a common cause of this pathology [13]. The diagnosis of AS resulting in severe aortic insufficiency and aortitis was confirmed according to clinical history, data of negative rheumatologic serologies test, echocardiography, and histological signs.

Interestingly, our patient had the history of reactive arthritis ten years ago, and HLA-B27 was detected at that time. He was diagnosed with AS four years later. The patient did not visit the rheumatologist for regular consultations, so his disease was poorly controlled, which led to cardiac pathology. The importance of proper treatment was confirmed in a 10-year population-based retrospective cohort study which showed, that sulfasalazine at its optimal daily dose reduces the risk of cardiovascular diseases in patients with AS [14]. It is also important that we assume that potentially more intensive anti-inflammatory treatment of active ankylosing spondylitis may have prevented the rapidly evolving LAD stenosis.

Another interesting finding was that our patient had an AS exacerbation and stenosis of the LAD after surgery with CPB. AS has been demonstrated as a risk factor for coronary artery disease. The pathophysiologic mechanism between these two diseases is chronic inflammation which causes endothelial dysfunction and direct endovascular injury resulting in atherosclerosis. On the contrary, inflammation of aortic wall (location is close to LAD) was observed during operation and was confirmed by pathohistological investigation (nonspecific aortitis). Furthermore, chronic inflammation causes hypercoagulable state [15]. Cardiac surgery using CPB is associated with inflammation [15]. We suggest that this could cause exacerbation of AS and development of coronary artery disease for our patient.

It is also important to think about percutaneous aortic valve implantation (TAVI) in situations like that. According to last ESC guidelines, the choice of the intervention mode surgical aortic valve replacement or TAVI should take into account the cardiac and extracardiac characteristics of the patient, the individual risk of surgery, which is assessed by the Heart Team, the feasibility of TAVI, and the local experience [16]. Data on TAVI are still very limited for patients younger than 75 years [17]. It has to be emphasized that younger patients differ with regard to anatomy (more bicuspid valves), which affects the results of TAVI (bicuspid valves were also in general excluded in clinical trials), and that long-term durability data for TAVI prosthetic valves are still lacking [17]. This means that, for older patients, TAVI could be really good decision in similar situations.

## 4. Conclusion

Poor control of AS may speed up the development of serious cardiac valve problems, requiring surgery with CPB. Cardiac operation with CPB may exacerbate the course of SA, in which symptoms are very similar to the life-threatening valve prosthesis-related infection. Inflammation in aortic root and hypercoagulable state could lead to narrowing of proximal parts of coronary arteries. Our case highlights the importance of SA control not only before cardiac operation but also immediately after it.

## Figures and Tables

**Figure 5:**